# Association between maternally inherited deafness, epilepsy, and intellectual disability and the m.12207G > A *MT-TS2* pathogenic variant in a Japanese family

**DOI:** 10.1016/j.ymgmr.2023.100966

**Published:** 2023-03-17

**Authors:** Sayaka Suzuki-Ajihara, Megumi Saito-Tsuruoka, Hiroko Harashima, Katsumi Arai, Hiroyoshi Koide, Yukiko Yatsuka, Atsuko Imai-Okazaki, Yasushi Okazaki, Kei Murayama, Chikahiko Numakura, Yuko Akioka, Akira Ohtake

**Affiliations:** a Department of Pediatrics, Saitama Medical University, Saitama, Japan; b Department of Clinical Genomics, Saitama Medical University, Saitama, Japan; c Hello Clinic, Saitama, Japan; d Diagnosis and Therapeutics of Intractable Diseases, Intractable Disease Research Center, Juntendo University Graduate School of Medicine, Tokyo, Japan; e Department of Metabolism, Chiba Children's Hospital, Chiba, Japan; f Center for Intractable Diseases, Saitama Medical University Hospital, Saitama, Japan

**Keywords:** M.12207G > A *MT-TS2*, Deafness, Epilepsy, Diabetes mellitus, Maternal inheritance

## Abstract

The identification of the m.12207G > A variant in *MT-TS2,* (NC_012920.1:m.12207G > A) was first reported in 2006. The affected individual presented with developmental delay, feeding difficulty, proximal muscle weakness, and lesions within her basal ganglia, with heteroplasmy levels of 92% in muscle and no evidence of maternal inheritance. Herein, we report a case involving a 16-year-old boy with the same pathogenic variation and different phenotype, including sensorineural deafness, epilepsy, and intellectual disability, without diabetes mellitus (DM). His mother and maternal grandmother had similar but milder symptoms with DM. Heteroplasmy levels of the proband in blood, saliva, and urinary sediments were 31.3%, 52.6%, and 73.9%, respectively, while those of his mother were 13.8%, 22.1%, and 29.4%, respectively. The differences in the symptoms might be explained by the different levels of heteroplasmy. To our knowledge, this is the first familial report of the m.12207G > A variant in *MT-TS2* that causes DM. The present case showed milder neurological symptoms than did the former report, and suggests the presence of a good phenotype–genotype correlation within this family.

## 1 Introduction

The human mitochondrial genome encodes only 13 proteins, two ribosomal RNAs, and 22 mitochondrial transfer RNAs. The tRNA genes are involved in the synthesis of proteins in mitochondria, with tRNA mutations resulting in mitochondrial dysfunction. The most commonly described mutation is the m.3243A > G *MT-TL1* point mutation. This mutation can give rise to different conditions, including mitochondrial myopathy, encephalopathy, lactic acidosis, and stroke-like episodes (MELAS), diabetes mellitus (DM), neurosensory deafness, pregnancy-induced hypertension, and progressive external ophthalmoplegia [1].

There are two types of tRNA^Ser^ (*MT-TS*): tRNA^Ser(UCN)^ (*MT-TS1*), for which the pathogenic variation are associated with hearing impairments, and tRNA^Ser(AGY)^ (*MT-TS2*). Although 10 variants have been reported for *MT-TS2*, only the m.12258C > A variant has been classified as “confirmed” (MITOMAP website (http://www.mitomap.org)) [[2], [3], [4]]. The presence of this pathogenic variation is associated with diabetes, retinitis pigmentosa, progressive sensorineural hearing loss, cataracts, and cerebellar ataxia.

By contrast, a previous study reported that the m.12207G > A variant is associated with mitochondrial myopathy and encephalopathy [5].

Here, we report a case found to have epilepsy, deafness, and developmental delay who was subsequently found to have the m.12207G > A variant.

## 2 Case report

Non-consanguineous Japanese parents who had undergone infertility treatment gave birth to their first child (male; birth weight, 3110 g), for whom the Apgar score was 3 at the first minute and 9 at the fifth minute. Motor and developmental milestones were almost normal. He was found to have sensory deafness at age 10 years and fitted with a hearing aid at age 12 years. Due to an intellectual disability, he began attending a school for disabled persons. The Wechsler Intelligence Scale for Children-IV was administered at age 13 years. The results were 55 for the overall score, 64 for the Verbal Comprehension Index, 66 for the Perceptual Reasoning Index, 65 for the Working Memory Index, and 55 for the Processing Speed Index. At age 16 years, he experienced a generalized clonic seizure for 50 s following a bout of vertigo. A computed tomography scan revealed calcification at the bilateral nucleus ganglia. Magnetic resonance imaging (MRI)/angiography, blood tests, and a neurological examination were unremarkable. His serum lactate level was 2.31 mM (< 2.1). Electroencephalography showed spikes at the frontal lobes. Due to the occurrence of subsequent muscle spasms, his medication was changed to levetiracetam (LEV). As his mother and maternal grandmother had similar symptoms, he was referred to our hospital for evaluation of mitochondrial disease.

**Fig. 1:**
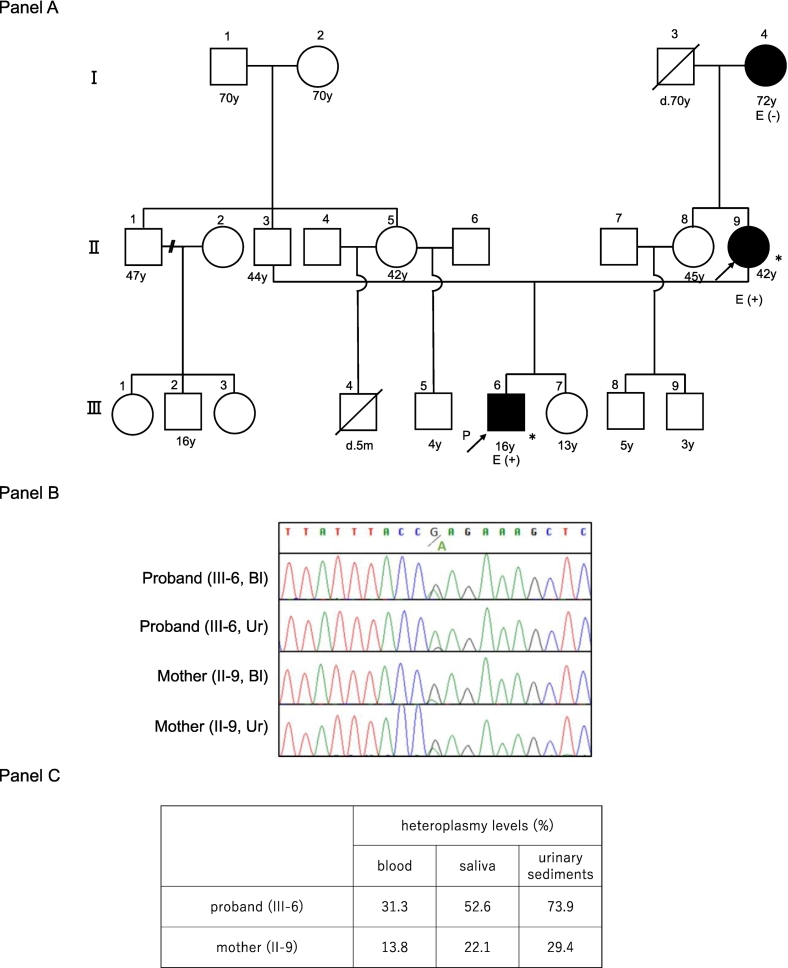
Panel A shows the pedigree. The black arrow is the proband. Panel B shows an electropherogram of Sanger sequencing containing m.12207G > A. Only the results of blood and urinary sediments are shown, although saliva was also analyzed. Panel C shows the proband and mother's heteroplasmy levels of blood, saliva, and urinary sediments. The heteroplasmy levels are higher in the proband than in his mother. Bl; blood, Ur: urinary sediments.

Family history (Fig. 1A).

The proband's mother (II-9) was diagnosed with epilepsy at age 13 years and had been taking LEV. At age 18 years, she was diagnosed with sensory deafness. She was subsequently fitted with a hearing aid for her left ear at age 19 years and for her right ear at age 23 years. DM was diagnosed at age 41 years. She presented with headache and vertigo at age 33 years, and an MRI T2-weighted image revealed the presence of hyperintense lesions in her bilateral nucleus ganglia and thalami.

Moreover, the proband's maternal grandmother (I-4) had been experiencing headaches since her 40s, accompanied by deafness and DM since her 50s. Both his mother and grandmother had intellectual disabilities.

The proband and his mother underwent an investigation of variants using a targeted panel of 367 nuclear genes known to cause mitochondrial diseases, as well as the whole mitochondrial genome [6]. Following the detection of the m.12207G > A variant in their blood and urinary sediments, Sanger sequencing was performed for confirmation (Fig. 1B). No other known pathogenic variants on mitochondrial DNA or nuclear DNA were detected. The m.12207G > A variants in blood, saliva, and urinary sediments were subjected to Sanger sequencing. Polymerase chain reaction products were directly sequenced using BigDye v3.1 Termination and ABI 3130XL (Applied Biosystems). Heteroplasmy levels were obtained from DNA sequencing analysis using the Minor Variant Finder software (Thermo) [7]. Those of the proband in the blood, saliva, and urinary sediments were 31.3%, 52.6%, and 73.9%, respectively, while those of the mother were 13.8%, 22.1%, and 29.4%, respectively (Fig. 1C).

Table 1 shows a summary of the clinical presentation of four patients (three from the present study and one previously reported). The family in this study presented with epilepsy, deafness, and DM, which differed from the previously reported patient.

**Table 1:** Clinical presentation.

	Proband (III-6)	Mother (II-9)	Grandmother (I-4)	Previous report [5]
Deafness	+	+	+	−
Epilepsy	+	+	−	−
Diabetes mellitus	−	+	+	−
Intellectual disability	+	+	+	+
Feeding difficulty	−	−	−	+
Muscle weakness	−	−	−	+
Basal ganglion involvement on MRI or CT	+	+	Untested	+

MRI: magnetic resonance imaging, CT: computed tomography.

## 3 Discussion

The present report evaluated inherited deafness along with epilepsy, intellectual disability, and DM caused by the m.12207G > A *MT-TS2* variant. The clinical heterogeneity seen in our patients was probably due to differences in heteroplasmy levels.

The m.12207G > A variant in the *MT-TS2* gene was first reported in a female patient who had complex I deficiency with developmental delay, feeding difficulty, proximal muscle weakness, lesions within the basal ganglia, cerebral atrophy, proximal muscle weakness, increased blood lactate, liver dysfunction, and fatty infiltration in muscle. The m.12207G > A variant in the previously described case was found in a heteroplasmic state (92%) within the skeletal muscle. Because it was not present in her unaffected mother's blood or in 200 healthy controls, the variant in the previous report might have occurred *de novo* [5].

The m.12207G > A variant occurs at the first nucleotide of the 5′ end of the *MT-TS2* gene, which is involved in the formation of the stem region of the amino acid acceptor arm. It may also affect the proper processing of the polycistronic RNA precursor [5,8]. Considering these facts, *MT-TS2* (NC_012920.1:m.12207G > A) is evaluated as “reported” in MITOMAP (https://www.mitomap.org/foswiki/bin/view/MITOMAP/MutationsRNA), but as “pathogenic” both in OMIM (https://www.omim.org/entry/590085?search=MT-TS2&highlight=mt-ts2) and ClinVar (https://www.ncbi.nlm.nih.gov/clinvar/variation/9561/?new_evidence=true).

The symptoms of the present patients differed from those in the previously described patient. In particular, our patients exhibited both deafness and epilepsy. In addition, the mother and grandmother had DM and were experiencing headaches. Whittaker et al. [9] reported the prevalence and progression of DM in mitochondrial disease. They described that the highest prevalence of DM was observed in the m.12258C > A group. M.12207G > A and m.12258C > A are both in *MT-TS2*. In the present family, both the proband's mother and grandmother had DM, and their onset ages were earlier than that reported in general, such as in the m.12258A > C variant [9].

Symptoms such as sensory deafness, developmental delay, and seizure were observed earlier in the proband than in his mother. The proband himself has not yet exhibited DM. However, the pathogenic variation loads in all three tissue samples were higher than those in the corresponding samples from his mother. Careful follow-up of his course over time is therefore necessary.

In this case, we did not evaluate the variant load in muscle because muscle biopsies are invasive, and many previous reports have shown that heteroplasmy levels in urine sediments were similarly or more useful than the level in skeletal muscle [[10], [11], [12]]. Fayssoil et al. [13] reported that the clinical expression of the disease in MELAS patients, including brain and heart involvement, correlates better with the m.3243A > G mutation load in urinary sediments than in other tissues. DNA from urinary sediments had the highest proportion of mutant genomes, whereas blood had the lowest, because of its slower turnover compared with blood cells. Therefore, urinary sediments are the source of choice when diagnosing mtDNA mutations, as they are easier to obtain, and the mutation load is almost always greater than that observed in the blood [10].

Shanske et al. [14] reported that cheek mucosa was another tissue of choice for the diagnosis of mtDNA pathogenic variants, so we also analyzed the heteroplasmy levels in saliva. The results showed that saliva was equally useful as urinary sediments. As the role of variation loads in saliva and urinary sediments may be almost equal to that in muscle, and always greater than that in blood, we think the pathogenicity of m.12207G > A in this family is confirmed.

In conclusion, this is the first known report to show that the m.12207G > A pathogenic variant can be maternally inherited and causes milder neurological symptoms than those cited in the former report. When evaluating mitochondrial disease, analysis of the m.12207G > A pathogenic variant in urinary sediments and saliva should be taken into consideration.

## Author contributions

SS-A and AO wrote the manuscript; MS-T and HH performed the sampling and data acquisition; KA and HK recruited the patients, provided the clinical information, and collected the samples; YY, AI-O, YO, and KM performed the genetic testing; and CN, YA, and AO supervised the project. All authors read and approved the final manuscript.

## Ethics

All procedures in the present study adhered to the ethical standards of the responsible committee on human experimentation and the Helsinki Declaration of the World Medical Organization. This study was approved by the Ethics Committee of Saitama Medical University (No. 844-VI), and written informed consent was obtained from all family members.

## Funding information

This work was supported by JSPS KAKENHI (grant No. JP20K08497) and the 10.13039/100009619Japan Agency for Medical Research and Development (AMED) (grant Nos. JP22ek0109468, JP19ek0109273, JP20kk0305015, and JP21ek0109495s0101).

## Data Availability

Data will be made available on request.
